# miR-155 Predicts Long-Term Mortality in Critically Ill Patients Younger than 65 Years

**DOI:** 10.1155/2019/6714080

**Published:** 2019-02-24

**Authors:** Frank Tacke, Martina E. Spehlmann, Mihael Vucur, Fabian Benz, Mark Luedde, David Vargas Cardenas, Sanchari Roy, Sven Loosen, Hans-Joerg Hippe, Norbert Frey, Christian Trautwein, Alexander Koch, Christoph Roderburg, Tom Luedde

**Affiliations:** ^1^Department of Medicine III, University Hospital RWTH Aachen, Pauwelsstrasse 30, 52074 Aachen, Germany; ^2^Department of Internal Medicine III, University of Kiel, Schittenhelmstrasse 12, 24105 Kiel, Germany

## Abstract

**Introduction:**

Alterations in miR-155 serum levels have been described in inflammatory and infectious diseases. Moreover, a role for miR-155 in aging and age-related diseases was recently suggested. We therefore analyzed a potential age-dependent prognostic value of circulating miR-155 as a serum-based marker in critical illness.

**Methods:**

Concentrations of circulating miR-155 were determined in 218 critically ill patients and 76 healthy controls.

**Results:**

By using qPCR, we demonstrate that miR-155 serum levels are elevated in patients with critical illness when compared to controls. Notably, levels of circulating miR-155 were independent on the severity of disease, the disease etiology, or the presence of sepsis. In the total cohort, miR-155 was not an indicator for patient survival. Intriguingly, when patients were subdivided according to their age upon admission to the ICU into those younger than 65 years, lower levels of miR-155 turned out as a strong marker, indicating patient mortality with a similar accuracy than other markers frequently used to evaluate critically ill patients on a medical ICU.

**Conclusion:**

In summary, the data provided within this study suggest an age-specific role of miR-155 as a prognostic biomarker in patients younger than 65 years. Our study is the first to describe an age-dependent miRNA-based prognostic biomarker in human diseases.

## 1. Introduction

Sepsis represents a complex pathological process including inflammation, coagulopathy, and deterioration of the patients' hemodynamic state, finally leading to organ failure [1]. Although it was shown that an immediate initiation of anti-infective and supportive therapeutic measures considerably improves the prognosis in critically ill patients [2], the overall sepsis-related mortality remains high. This highlights the need for biomarkers allowing an early possible diagnosis on the one hand and prognosis assessment to guide therapy, on the other [3, 4].

MicroRNAs represent endogenous RNA molecules with a length of ~22 nucleotides [5]. They are created by a complex process leading from pre-miRNAs to the mature miRNA that regulates multiple processes such as cell metabolism, cell growth, and differentiation as well as cell death [5]. miR-155 represents one of the best characterized microRNAs in the context of infection and inflammation. This miRNA is predominantly found in the liver, spleen, and thymus [6] and is involved in immune cell development and the regulation of systemic inflammatory processes [7–9]. Alterations in miR-155 expression were demonstrated in activated immune cells and consequently in many inflammatory diseases such as allergic asthma [8], atopic dermatitis [10], rheumatoid arthritis [11], Crohn's disease [12], and liver injury [13]. In a recently published *in vitro* study using LPS-induced THP-1 monocytes, it was demonstrated that miR-155 regulated the expression of different proinflammatory mediators [14], arguing for a function in systemic inflammation and infection.

Due to their simple chemical structure and the resulting biological stability, circulating miRNAs were proposed by many authors as biomarkers for several diseases including inflammatory diseases and infections [15]. In particular, many authors proposed circulating miRNAs as serum-based markers in patients with critical illness. Nevertheless, despite intensive research efforts, specific mechanisms or pathogenic factors regulating concentrations of circulating miRNAs in sepsis (and many other diseases) are poorly understood.

Here, we analyzed the diagnostic and prognostic value of miR-155 serum levels in 218 critically ill patients treated on an intensive care unit.

## 2. Methods

### 2.1. Study Design and Patient Characteristics

Between 2010 and 2013, 218 patients (see Table 1), consecutively admitted to the Internal Medicine Intensive Care Unit at the University Clinic (RWTH) Aachen, were included. Patients, expected having an intensive care treatment < 3 days, were excluded. After discharge, patients were included into a follow-up by contacting the patients, the patients' relatives, or the primary care physician. Sepsis, severe sepsis, and septic shock were diagnosed based on the criteria published by the ACCP/SCCM Consensus Conference. 76 healthy blood donors (47 males, 29 females; median age 33 years, range 18-67) with normal values for blood counts, C-reactive protein, and liver enzymes served as a control as recently described [16].

### 2.2. miRNA Isolation [16, 17]

400 *μ*l serum was spiked with miScript miRNA mimic SV40 (Qiagen 2 *μ*M, 1 *μ*l/100 *μ*l serum). 800 *μ*l phenol (Qiazol) and 200 *μ*l chloroform were added to the sample and mixed vigorously for 15 sec followed by an incubation at room temperature for 10 min, followed by centrifugation for 15 min at 12,000 g. The aqueous phase was precipitated with 500 *μ*l 100% isopropanol and 2 *μ*l glycogen (Fermentas, St. Leon-Rot, Germany) o.n. at -20°C. After centrifugation at 4°C for 30 min (12,000 g), the pellets were washed with 70% EtOH and RNA was resuspended in 30 *μ*l RNase-free water (Ambion, Austin, TX). RNA quality was assessed using a NanoDrop spectrophotometer (NanoDrop), and a small RNA assay for Agilent's Bioanalyzer was performed (Agilent Technologies, Böblingen, Germany).

### 2.3. Quantitative Real-Time PCR [16, 17]

5 *μ*l of extracted total RNA was used for cDNA synthesis using the miScript Reverse Transcriptase Kit (Qiagen). cDNA samples were used for qPCR using the miScript SYBR Green PCR Kit (Qiagen) and miRNA-specific primers (Qiagen) on a qPCR machine (Applied Biosystems 7300 Sequence Detection System, Applied Biosystems, Foster City, CA). Data were generated and analyzed using the SDS 2.3 and RQ manager 1.2 software packages.

### 2.4. Statistical Analysis [16–19]

Data are given as the median and range using the Mann-Whitney *U* test, and for multiple comparisons, the Kruskal-Wallis *H* test was used. Box plot graphics display a statistical summary of the median, quartiles, and ranges. Correlation analyses were performed by using the Spearman correlation tests. Kaplan-Meier curves were used to analyze the overall survival (OS). Optimal cut-off values were established using the well-established Youden index as described before. The prognostic relevance of serum miR-155 was further tested using univariate Cox regression analysis. ROC curves were generated by plotting sensitivity against 1 − specificity. All statistical analyses were performed with SPSS (SPSS 23, Chicago, IL, USA).

## 3. Results

### 3.1. miR-155 Serum Levels Are Elevated in Critically Ill Patients

**Figure 1:**
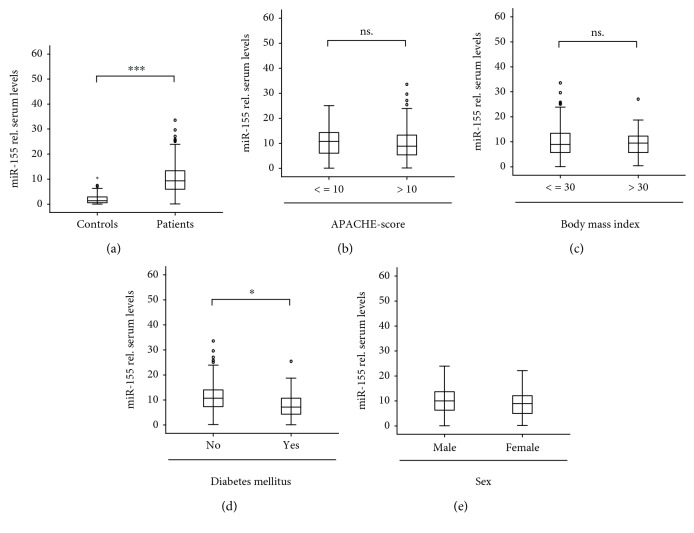
Serum miR-155 levels of critically ill patients at ICU admission. (a) qPCR was used to determine the concentrations of circulating miR-155 at admission to the ICU. In this analysis, critically ill patients (*n* = 218) displayed significantly higher serum levels of miR-155 compared to healthy controls (*n* = 76). (b) Serum miR-155 concentrations were independent on disease severity. (c) Serum concentrations of miR-155 were measured in patients with/without diabetes mellitus type 2. (d) Serum concentrations of miR-155 independent on the presence of obesity. (e) Serum concentrations of miR-155 did not vary with respect to patients' sex. ^∗∗∗^ *p* < 0.001.

We measured miR-155 serum concentrations in 218 patients upon admission to the ICU and in 76 healthy controls. In these analyses, miR-155 serum concentrations were significantly elevated in critically ill patients (Figure 1(a)). Next, we analyzed whether miR-155 concentrations might reflect the disease severity in critically ill patients. Therefore, we subdivided our cohort of patients into those with a more severe disease state versus a less severe disease state according to APACHE-II score values. Interestingly, both groups displayed similar miR-155 serum concentrations (Figure 1(b)), indicating that circulating miR-155 is independent of the disease severity in critically ill patients.

It was recently demonstrated that metabolic comorbidities determine the prognosis and treatment outcome of patients treated on a medical ICU. As alterations in miR-155 serum levels have recently been found in metabolic diseases, we next analyzed the impact of preexisting type 2 diabetes or obesity on miR-155 concentrations. Of note, we found significantly lower levels of miR-155 in patients with type 2 diabetes, while miR-155 concentrations were independent on the presence of obesity (Figures 1(c) and 1(d)).

There is increasing evidence for sex differences in inflammatory pathologies. Therefore, we analyzed the levels of circulating miR-155 specifically in male and female patients. Notably, no differences were found in this analysis (Figure 1(e)).

### 3.2. miR-155 Serum Levels Are Not Affected by the Presence of Sepsis

Based on recent results, suggesting that mir-155 serum concentrations are elevated in sepsis [20], we subdivided our patients into those that fulfilled sepsis 3 criteria (*n* = 135) and those that did not (patients' characteristics are given in Table 2). Interestingly, no significant differences in circulating miR-155 levels between both subgroups of patients became apparent (Supplementary Figure 1A). To further substantiate this finding, we performed correlation analyses between miR-155 and parameters routinely used to access the presence of sepsis in critically ill patients. In this analysis, serum miR-155 levels were not correlated to pro- or anti-inflammatory C-reactive protein (CRP; *r* = 0.027, *p* = 0.770), procalcitonin (PCT; *r* = 0.133, *p* = 0.197), interleukin-6 (IL-6; *r* = 0.084, *p* = 0.519), interleukin-10 (IL-10; *r* = −0.002, *p* = 0.983), or tumor necrosis factor (TNF; *r* = −0.022, *p* = 0.990). Next, we hypothesized that miR-155 serum levels might be altered in specific disease etiologies. However, the differences between the various etiologies did not meet the criteria of statistical significance (Supplementary Figure 1B).

### 3.3. miR-155 Serum Concentrations Predict Survival Specifically in Critically Ill Patients Younger than 65 Years

**Figure 2:**
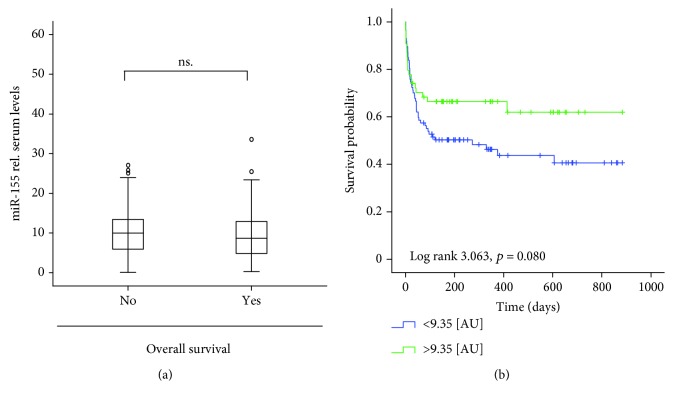
Serum levels of miR-155 are not predictive for patients' overall prognosis. (a) Serum levels of miR-155 were analyzed by qPCR in critically ill patients that survived in the long-term follow-up or succumbed to death. No difference between these groups became apparent. (b) Patients with miR-155 levels below or higher than the median of all patients displayed a similar long-term survival.

We next tested whether miR-155 serum levels might allow to estimate overall mortality. We therefore compared miR-155 levels in patients that succumbed to death to those in patients that survived in the long-term follow-up. However, no differences between both patient groups became apparent (Figures 2(a) and 2(b)).

**Figure 3:**
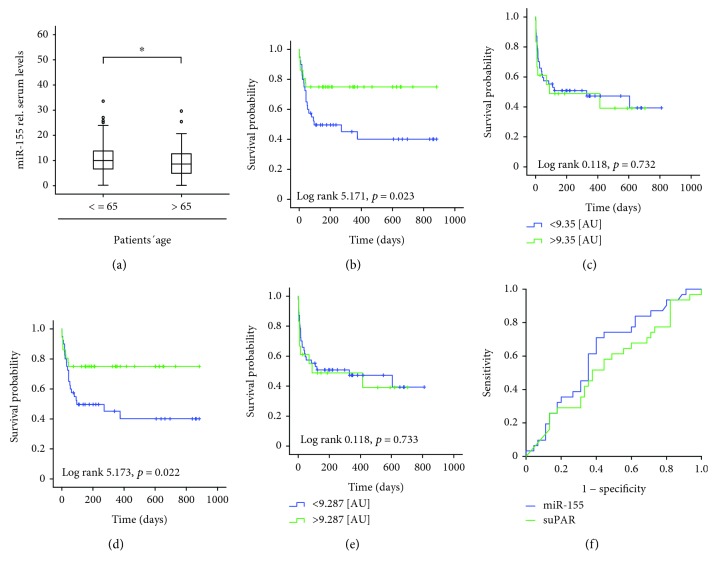
Serum concentrations of ICU predict long-term survival specifically in young ICU patients. (a) miR-155 serum levels in patients younger or older than 65 years. (b, c) Kaplan-Meier curve analysis demonstrating that patients < 65 years (but not older patients) with miR-155 concentrations below the median of all patients had an increased overall mortality. (d) The Youden index was used to calculate the optimal threshold for distinguishing between long-term survivors and patients that did not survive in the group of patients < 65 years old. Kaplan-Meier survival curve analyses revealed that patients with miR-155 concentrations below this threshold had an increased overall mortality. (e) Kaplan-Meier curve analysis was performed in patients > 65 years old, revealing that the mortality of these patients was independent of their miR-155 serum concentration. (f) ROC curve analysis revealing that miR-155 serum levels display a superior prognostic value in critically ill patients younger than 65 years. ^∗^ *p* < 0.05.

Based on the recent data suggesting that the expression patterns and functions of miRNAs might depend on the patients' age, we next compared concentrations in circulating miR-155 between patients that were older than 65 years on those that were younger at the time point of admission to the ICU. Interestingly, we found statistically significant lower miR-155 levels in the subgroup of the older patients compared to patients < 65 years old (Figure 3(a); patients' characteristics are given in Supplementary Figure 2, Tables 1 and 2). In line, miR-155 levels correlated with patients' age (*r* = −0.212; *p* = 0.002). To evaluate the impact of age on the role of miR-155 as a prognostic biomarker in critical illness, we performed Kaplan Meier curve analysis separately in the subgroup of the younger (<65 years) and the older (>65 years) patients. Strikingly, while in the subgroup of patients < 65 years, those with low miR-155 concentrations (below the median of all patients) displayed a significantly impaired overall mortality compared to the other patients (Figure 3(b)), no such effect was observed in the group of the patients > 65 years (Figure 3(c)). To substantiate these findings, the Youden index was used to determine an optimal threshold of miR-155 levels for predicting patients' survival within the group of the patients < 65 years [21]. This analysis revealed that relative miR-155 concentrations of 9.28 (AU) had the best sensitivity and specificity to decide whether a patient will survive or not. Strikingly, patients with miR-155 serum concentrations >9.28 (AU) demonstrated a significantly longer survival compared to patients with lower values (Figures 3(c) and 3(d)). Again, no such effect was seen in the subgroup of the critically ill patients older than 65 years (Figure 3(e)). Consequently, circulating miR-155 demonstrated a significant correlation with the patients' survival time specifically in the group of the young ICU patients (*r* = 0.443, *p* = 0.001 vs. *r* = 0.170, *p* = 0.330) and differences in mortality between patients with low (51%) vs. high (29%, *p* = 0.014) serum concentrations of miR-155 were only apparent in this specific collective of patients, while in older patients a similar mortality was observed (56% vs. 62%, n.s.).

Finally, we used ROC curve analysis, showing that the prognostic value of miR-155 is similar to that of the APACHE-II score, patient's age, serum creatinine concentration, INR, and suPAR (Figure 3(f); Supplementary Figure 3). In summary, these results imply a novel function of miR-155 serum levels as a prognostic serum-based marker that is only apparent in critically ill patients younger than 65 years.

## 4. Discussion and Conclusions

miR-155 levels were recently described as diagnostic biomarkers in coronary artery disease [22, 23] and dissection of the ascending aorta [24] and have also been validated as a powerful biomarker in B-cell malignancies [25], esophageal cancer [20], and other malignant diseases [26]. However, studies proving a diagnostic or prognostic role of miR-155 in critical illness are not available yet. Our study reports a novel role of circulating miR-155 in distinguishing critically ill patients from healthy controls and in predicting survival of critically ill patients < 65 years old. Our data rely on a large sequentially recruited cohort comprising 218 critically ill patients that were precisely characterized regarding clinical characteristics. 76 healthy blood donors that were unfortunately not age-matched to the patients were used as controls.

A Chinese study group investigated a cohort of sixty patients and found that, compared to healthy controls, sepsis patients exhibit significantly elevated miR-155 levels, which is positively related to a greater severity of sepsis [20]. Neither could we identify an influence of sepsis on miR-155 levels nor were miR-155 serum levels correlated to disease severity. Interestingly, elevated miR-155 levels were predictive of a severe condition and poor prognosis in sepsis patients [20]. In accordance, our study identifies the prognostic value of miR-155 regarding survival. However, we demonstrate that low miR-155 levels correlate with reduced survival in young critically ill patients including the group of patients with sepsis.

In the pathophysiology of critical illness, life-threatening diseases of diverse origins like infection and shock lead to instant local and systemic physiologic responses involving all major organs [27]. The time course and severity of the host response to damage are determined by both the intensity of the trauma and host factors, and the activation of the first-line inflammatory immune response is sometimes followed by immunosuppression. Prior studies identified miR-155 as an integral part of the initial immune reaction (i.e., activation of macrophages) to diverse inflammation-causing agents. For example, it was shown that miR-155 expression can be initiated upon stimulation with LPS in a human monocytic cell line [28]. Furthermore, activators of inflammation like interferon-*β* or TNF can provoke miR-155 expression in macrophages and monocytes. In patients with critical illness, the inflammatory mediator LPS is found in higher levels compared to healthy probands [29]. Moreover, LPS and TNF exert profound influence on the systemic inflammatory response caused by infection, trauma, burns or hemorrhagic shock, and pancreatitis [30, 31]. Investigations using on a mouse macrophage/monocyte cell line showed that an increase in TNF levels by LPS results in the upregulation of miR-155 [32]. Conversely, LPS-induced upregulation of miR-155 leads to increased TNF production [32]. Furthermore, studies in a transgenic mouse line overexpressing miR-155 in a B-cell lineage synthetize more TNF when stimulated with LPS and are highly susceptible to septic shock induced by LPS [32]. Thus, a possible pathomechanism in the pathogenesis of critical illness might be the interaction between miR-155 and LPS/TNF. One possible explanation might be that in patients with critical illness, increased LPS levels might lead to the upregulation of miR-155 which then leads to an increase in TNF, contributing to long-term detrimental effects on survival in these patients. It is conceivable that miR-155 raises TNF levels by improving transcript stability through binding to its 3′UTR. Finally, targeting and reduction of expression of SHIP1 phosphatase by miR-155 might explain its proinflammatory effects observed in different pathologies [33]. As another option, miR-155 could influence gene transcripts coding for proteins that are recognized for their ability to suppress TNF-*α* translation. This pathomechanism will be subject to studies in the future. Furthermore, miR-155 is reported to target and reduce the expression of genes involved in the LPS/TNF such as FADD. Therefore, it will be important to characterize further downstream targets of miR-155 in this context. We demonstrate a significant association of miR-155 levels with the presence of critical illness and miR-155 levels correlated with survival in patients younger than 65 years old. Several investigators have reported that the amount of serum miRNA is age dependent [34] and that circulating microvesicle number, function, and small RNA content vary with age [35]. Importantly, miR-155 has been identified to have a significantly lower abundance in peripheral blood mononuclear cells of older (mean age 65 years old) individuals [36]. These findings could explain the fact that the prognostic value of miR-155 levels is compromised with increased age.

In summary, our data and results from previous studies imply a novel function of miR-155 as a serum-based marker in critically ill patients.

## Figures and Tables

**Table 1:** Baseline patient characteristics.

Parameter	All patients	<65 years	>65 years
Number	218	125	93
Sex (male/female)	138/80	82/43	56/37
Age median (range) (years)	63 (18-89)	52 (18-65)	74 (66-89)
APACHE II score median (range)	17 (2-43)	15 (2-43)	19 (5-40)
SAPS2 score median (range)	43.0 (0-79)	40 (9-79)	45 (0-72)
ICU days median (range)	7 (1-83)	7 (1-70)	7 (1-83)
Death during ICU or follow-up (%)	47.2%	36%	51.6%
28 d mortality	24.8%	18.2%	31.3%
Ventilation time median (range) (h)	129 (0.5-1363)	127 (0.5-928)	132 (1-1363)
Diabetes mellitus (%)	30.7%	20.0%	45.16%
Body mass index (BMI)	26.78 (16.6-86.5)	26 (16.6-86.5)	26.12 (19.3-61)
Creatinine	1.3 (0-15)	1.3 (0.2-15)	1.35 (0-11.5)
Albumin	27.0 (15.2- 52.2)	26 (15.2-41)	28.6 (15.8-52.2)
WBC median (range) (×10^3^/*μ*l)	12.15 (0.1-67.4)	11.65 (0.1-67.4)	12.7 (0.1-66.2)
CRP median (range) (mg/dl)	95.5 (<5-230)	112 (5-230)	90 (<5-230)
Procalcitonin median (range) (*μ*g/l)	0.7 (0-180.6)	0.7 (0.06-125.2)	0.65 (0-180.6)
Interleukin-6 median (range) (pg/ml)	105 (0-83000)	130 (2-28000)	100 (0-83000)
Tumor necrosis factor median (pg/ml)	19 (4.9-140)	19 (4.9-140)	20 (10-100)
Serum lactate (mmol/l)	1.70 (0-21.9)	1.5 (0-21.9)	1.7 (0-20.8)
miR-155 median (range) (rel. ex.)	9.35 (0.1-56.49)	10.05 (0.17-56.49)	8.63 (0.1-29.65)

APACHE: acute physiology and chronic health evaluation; CRP: C-reactive protein; ICU: intensive care unit; SAPS: simplified acute physiology score; WBC: white blood cell count.

**Table 2:** Disease etiology of the study population.

	All patients	<65 years	>65 years
Sepsis critical illness	*n* = 135	*n* = 74	*n* = 61
*Source of infectionn(%)*			
Pulmonary	71	34	37
Abdominal	28	17	11
Urogenital	3	3	0
Other	33	20	12
Nonsepsis critical illness *n* (%)	*n* = 83	*n* = 51	*n* = 32
Cardiopulmonary disease	28	13	16
Decompensated liver cirrhosis	12	9	3
Nonsepsis other	43	29	14

## Data Availability

The data used to support the findings of this study are available from the corresponding author upon request.

## Abbreviations

ACCP/SCCM: American College of Chest Physicians and the Society of Critical Care Medicine
ALT: Alanine aminotransferase
APACHE: Acute physiology and chronic health evaluation
APRIL: A proliferation-inducing ligand
AST: Aspartate aminotransferase
AUC: Area under the curve
BMI: Body mass index
BNP: Brain natriuretic peptide
CRP: C-reactive protein
DNA: Deoxyribonucleic acid
GFR: Glomerular filtration rate
ICU: Intensive care unit
IL: Interleukin
INR: International normalized ratio
LDH: Lactate dehydrogenase
miRNA: Microribonucleic acid
PCHE: Pseudocholinesterase
PCR: Polymerase chain reaction
PCT: Procalcitonin
RNA: Ribonucleic acid
ROC: Receiver operating characteristic
SAPS: Simplified acute physiology score
SIRS: Systemic inflammatory response syndrome
SOFA: Sequential organ failure assessment
SuPAR: Soluble urokinase-type plasminogen activator receptor
TNF: Tumor necrosis factor
WBC: White blood cell count.

## Additional Points

*Key Messages*. miR-155 serum concentrations are elevated in critically ill patients; are independent on the severity of disease, the disease etiology, and the presence of sepsis; do not reflect the overall survival in the total cohort of patients; and predict long-term survival specifically in patients < 65 years old.

## Supplementary Materials

Supplementary Materials Supplementary Figure 1: serum miR-155 concentrations are unaltered in sepsis. (A) miR-155 serum levels were analyzed in patients with or without sepsis. (B) miR-155 was analyzed in different disease etiologies. Supplementary Figure 2: analysis of the cohort with respect to patients' age. (A) miR-155 serum levels were analyzed in patients < 65 years with different disease severities. (B) miR-155 serum levels were analyzed in patients < 65 years with or without sepsis. (C) miR-155 serum levels analyzed in patients < 65 years with different disease etiologies. (D) Serum concentrations of miR-155 were analyzed in patients < 65 years with or without diabetes mellitus type 2. (E) Serum concentrations of miR-155 were analyzed in patients < 65 years with or without obesity. (F) miR-155 serum levels were analyzed in patients > 65 years with different disease severities. (G) miR-155 serum levels were analyzed in patients > 65 years with or without sepsis. (H) miR-155 serum levels analyzed in patients > 65 years with different disease etiologies. (I) Serum concentrations of miR-155 were analyzed in patients > 65 years with or without diabetes mellitus type 2. (J) Serum concentrations of miR-155 were analyzed in patients > 65 years with or without obesity. Supplementary Figure 3. prognostic value of miR-155 serum levels in patients younger than 65 years old. ROC curve analysis was performed. Supplementary Table 1A: disease etiology of the study population (<65 years) Supplementary Table 1B: disease etiology population (>65 years).
