# Supplementary material for: miR-155 Predicts Long-Term Mortality in Critically Ill Patients Younger than 65 Years
Source: Mediators Inflamm. 2019 Feb 24;2019:6714080. doi: 10.1155/2019/6714080 (PMC6409014; doi:10.1155/2019/6714080)
Supplement: Supplementary Materials — Supplementary Figure 1: serum miR-155 concentrations are unaltered in sepsis. (A) miR-155 serum levels were analyzed in patients with or without sepsis. (B) miR-155 was analyzed in different disease etiologies. Supplementary Figure 2: analysis of the cohort with respect to patients' age. (A) miR-155 serum levels were analyzed in patients < 65 years with different disease severities. (B) miR-155 serum levels were analyzed in patients < 65 years with or without sepsis. (C) miR-155 serum levels analyzed in patients < 65 years with different disease etiologies. (D) Serum concentrations of miR-155 were analyzed in patients < 65 years with or without diabetes mellitus type 2. (E) Serum concentrations of miR-155 were analyzed in patients < 65 years with or without obesity. (F) miR-155 serum levels were analyzed in patients > 65 years with different disease severities. (G) miR-155 serum levels were analyzed in patients > 65 years with or without sepsis. (H) miR-155 serum levels analyzed in patients > 65 years with different disease etiologies. (I) Serum concentrations of miR-155 were analyzed in patients > 65 years with or without diabetes mellitus type 2. (J) Serum concentrations of miR-155 were analyzed in patients > 65 years with or without obesity. Supplementary Figure 3. prognostic value of miR-155 serum levels in patients younger than 65 years old. ROC curve analysis was performed. Supplementary Table 1A: disease etiology of the study population (<65 years) Supplementary Table 1B: disease etiology population (>65 years). [file 6714080.f1.pdf]

Tacke et al. Supplementary Figure 1

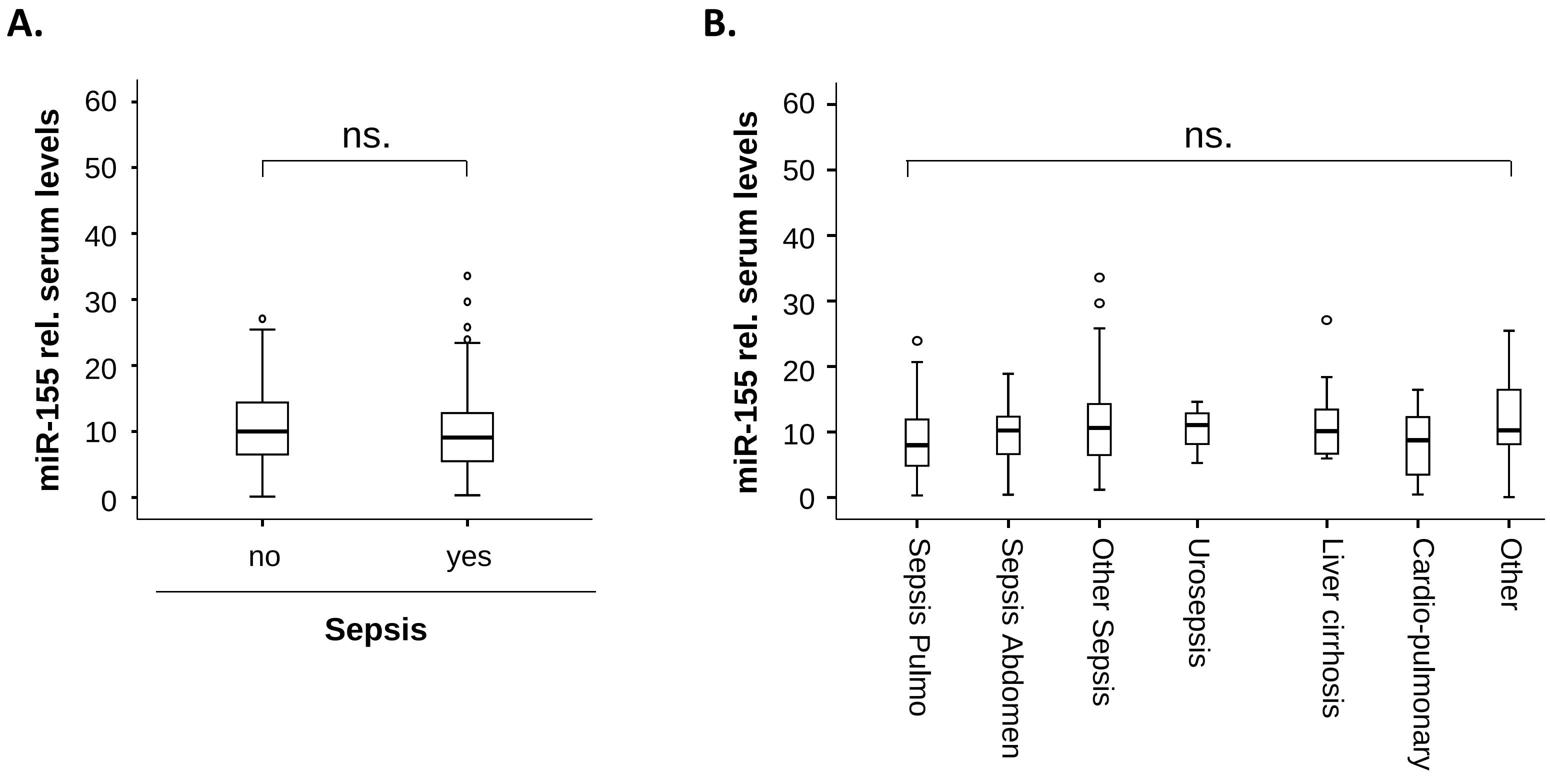

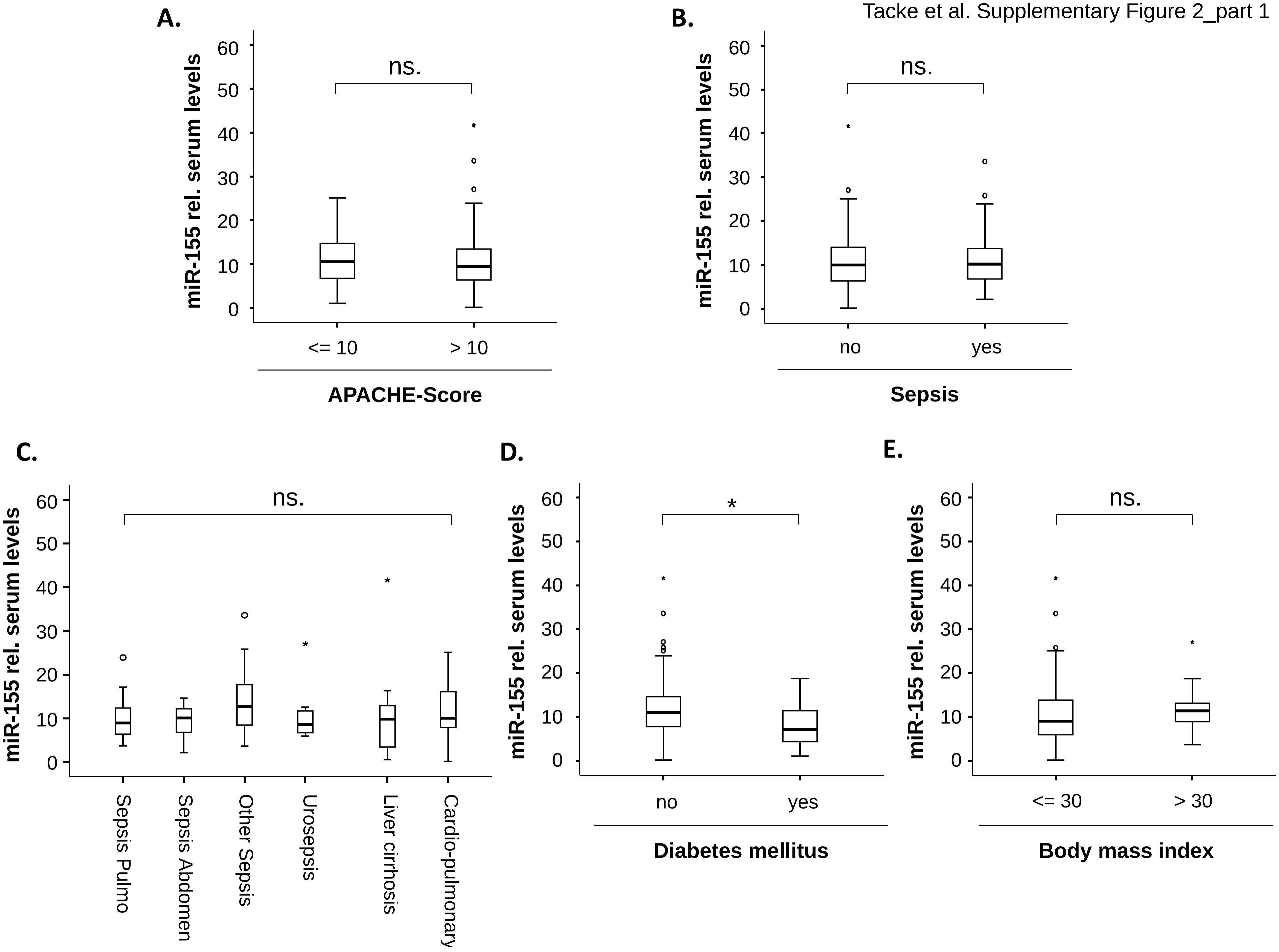
A.
miR-155 rel. serum levels
ns.
<= 10
> 10
APACHE-Score
B.
miR-155 rel. serum levels
ns.
no
yes
Sepsis
C.
miR-155 rel. serum levels
ns.
Sepsis Pulmo
Sepsis Abdomen
Other Sepsis
Urosepsis
Liver cirrhosis
Cardio-pulmonary
D.
miR-155 rel. serum levels
*
no
yes
Diabetes mellitus
E.
miR-155 rel. serum levels
ns.
<= 30
> 30
Body mass index

Tacke et al. Supplementary Figure 2_part 2

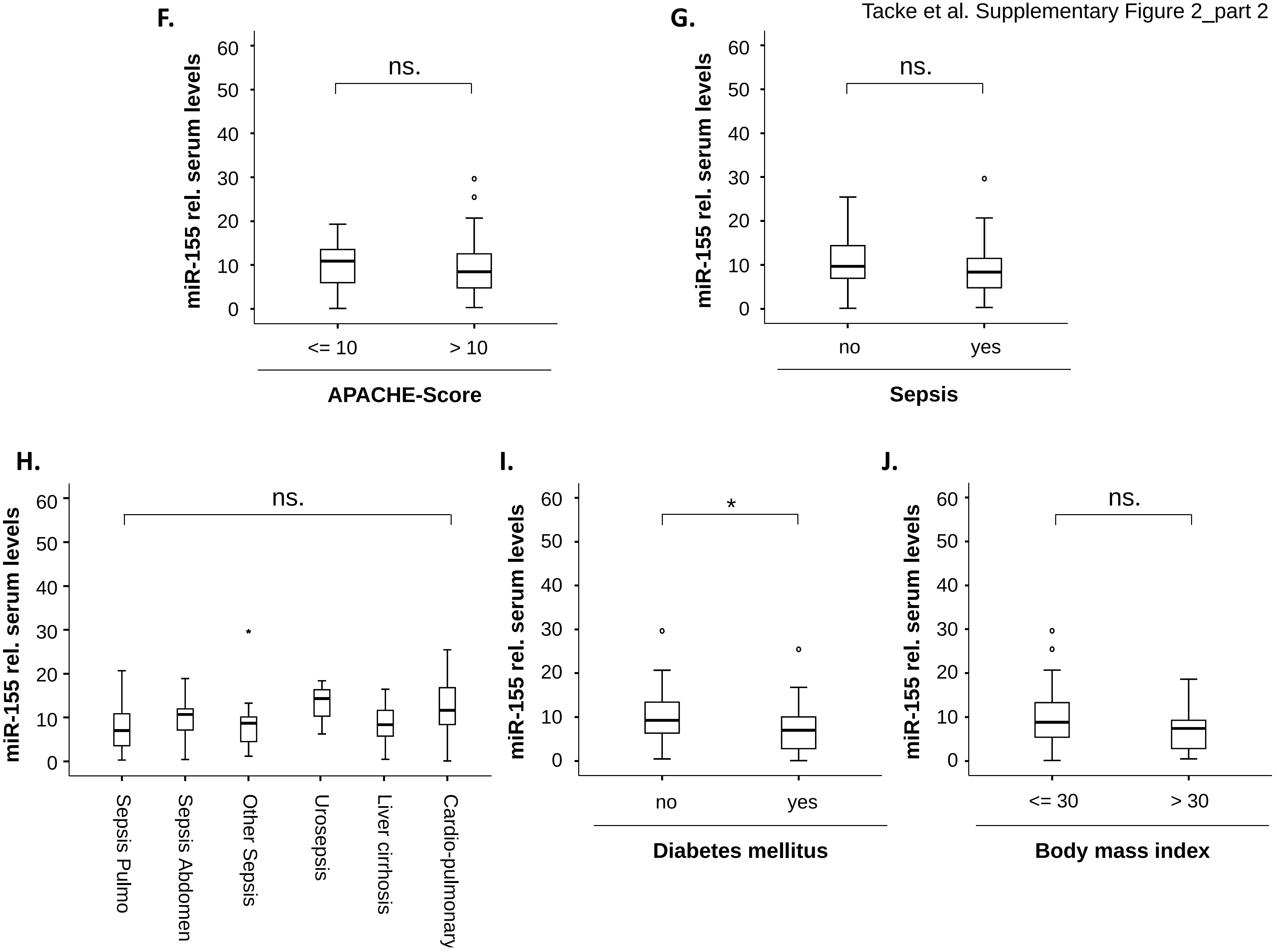

Tacke et al. Supplementary Figure 3

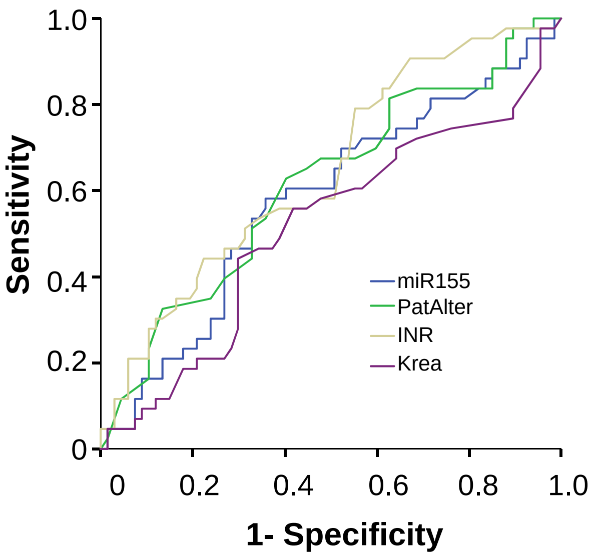

Supplementary Table 1A. Disease etiology of the study population (< 65 years)

	sepsis	non-sepsis
	n=74	n=51
**Sepsis critical illness**<br>Source of infection n (%)		
Pulmonary	34 (27.2%)	
Abdominal	17 (13.6%)	
Urogenital	3 (2.4%)	
Other	20 (16.0%)	
**Non-sepsis critical illness**<br>n (%)		
cardiopulmonary disease		13 (10.4%)
decompensated liver cirrhosis		9 (7.2%)
non-sepsis other		29 (23.2%)

Supplementary Table 1B. Disease etiology population (> 65 years)

	sepsis	non-sepsis
	n=60	n=33
**Sepsis critical illness**<br>Source of infection n (%)		
Pulmonary	37 (39.8%)	
Abdominal	11 (11.8%)	
Urogenital	0 (0%)	
Other	12 (12.9%)	
**Non-sepsis critical illness**<br>n (%)		
cardiopulmonary disease		16 (17.2%)
decompensated liver cirrhosis		3 (3.2%)
non-sepsis other		14 (15.1%)
